# Morphological and Behavioral Impact of AAV2/5-Mediated Overexpression of Human Wildtype Alpha-Synuclein in the Rat Nigrostriatal System

**DOI:** 10.1371/journal.pone.0081426

**Published:** 2013-11-27

**Authors:** Sara E. Gombash, Fredric P. Manfredsson, Christopher J. Kemp, Nathan C. Kuhn, Sheila M. Fleming, Ann E. Egan, Laura M. Grant, Michelle R. Ciucci, Jeffrey P. MacKeigan, Caryl E. Sortwell

**Affiliations:** 1 Graduate Program in Neuroscience, University of Cincinnati, Cincinnati, Ohio, United States of America; 2 Department of Translational Science and Molecular Medicine, Michigan State University, Grand Rapids, Michigan, United States of America; 3 Departments of Psychology and Neurology, University of Cincinnati, Cincinnati, Ohio, United States of America; 4 Departments of Surgery and Communications Sciences and Disorders, University of Wisconsin, Madison, Wisconsin, United States of America; 5 Laboratory of Systems Biology, Van Andel Research Institute, Grand Rapids, Michigan, United States of America; Emory University, United States of America

## Abstract

The discovery of the involvement of alpha-synuclein (α-syn) in Parkinson’s disease (PD) pathogenesis has resulted in the development and use of viral vector-mediated α-syn overexpression rodent models. The goal of these series of experiments was to characterize the neurodegeneration and functional deficits resulting from injection of recombinant adeno-associated virus (rAAV) serotype 2/5-expressing human wildtype α-syn in the rat substantia nigra (SN). Rats were unilaterally injected into two sites in the SN with either rAAV2/5-expressing green fluorescent protein (GFP, 1.2 x 10^13^) or varying titers (2.2 x 10^12^, 1.0 x 10^13^, 5.9 x 10^13^, or 1.0 x 10^14^) of rAAV2/5-α-syn. Cohorts of rats were euthanized 4, 8, or 12 weeks following vector injection. The severity of tyrosine hydroxylase immunoreactive (THir) neuron death in the SN pars compacta (SNpc) was dependent on vector titer. An identical magnitude of nigrostriatal degeneration (60-70% SNpc THir neuron degeneration and 40-50% loss of striatal TH expression) was observed four weeks following 1.0 x 10^14^ titer rAAV2/5-α-syn injection and 8 weeks following 1.0 x 10^13^ titer rAAV2/5-α-syn injection. THir neuron degeneration was relatively uniform throughout the rostral-caudal axis of the SNpc. Despite equivalent nigrostriatal degeneration between the 1.0 x 10^13^ and 1.0 x 10^14^ rAAV2/5-α-syn groups, functional impairment in the cylinder test and the adjusting steps task was only observed in rats with the longer 8 week duration of α-syn expression. Motor impairment in the cylinder task was highly correlated to striatal TH loss. Further, 8 weeks following 5.9 x 10^13^ rAAV2/5-α-syn injection deficits in ultrasonic vocalizations were observed. In conclusion, our rAAV2/5-α-syn overexpression model demonstrates robust nigrostriatal α-syn overexpression, induces significant nigrostriatal degeneration that is both vector and duration dependent and under specific parameters can result in motor impairment that directly relates to the level of striatal TH denervation.

## Introduction

Preclinical animal models that can accurately predict clinical efficacy and recapitulate the pathological aspects of Parkinson’s disease (PD) are a necessity. There continues to be substantial interest in models that overexpress the protein alpha-synuclein (α-syn) to model parkinsonism. A large body of evidence points to α-syn’s involvement in PD, including the fact that point mutations and multiplications of the *SNCA* gene have been linked to onset of familial forms of PD [1-3]. Subsequent discoveries of the presence of α-syn in the hallmark protein aggregations (Lewy Bodies) and dystrophic neurites of PD have linked α-syn to sporadic forms of the disease [4,5]. Despite evidence that α-syn may play a role in PD, the precise normal function of this widespread neuronal protein remains equivocal. However, it is understood that α-syn plays a functional role in synaptic vesicle release [6-9], vesicle trafficking [10], mitochondrial function [11-13], and can alter dopamine handling and synthesis [14-18], all of which may play a role in the PD disease process if α-syn levels are deregulated [19].

Recently, there has been increased use of the viral vector-mediated α-syn nigrostriatal overexpression rodent model of PD. These α-syn overexpression models recapitulate many of the neuropathological hallmarks of the human form of the disease, including but not limited to, α-syn aggregation, degeneration of nigral dopaminergic neurons and striatal terminals, and neuroinflammation. Both recombinant adeno-associated viral vectors (rAAV) and lentiviral vectors have been used to overexpress the human wildtype and mutated forms of α-syn in the rodent nigrostriatal system [20-26]. These α-syn overexpression studies have been useful in uncovering the relationship between α-syn protein expression and nigrostriatal neurodegeneration; however, the lack of standardized experimental parameters between studies have produced varying pathological and behavioral results. For example, variations in vector promoter and serotype, differences in genome copy numbers (titer), vector handling, and in number of injection sites and injection volume all influence transgene expression within nigral neurons. Previous studies have demonstrated that rAAV2/5-α-syn induces a progressive nigral dopamine neuron death over time, with the magnitude and time course of degeneration likely to depend on level of α-syn expression within individual neurons and number of infected nigral dopamine (DA) neurons [24,27]. 

rAAV-mediated α-syn overexpression resulting from the use of vector serotypes 2, 5, or 6 in the rat nigrostriatal system results in a wide range of 40-70% nigral DA neuron loss over a period of 8-27 weeks [20,23-30]. Behavioral deficits following α-syn overexpression have been inconsistent, likely due to the fact that not all rAAV-α-syn injections result in the magnitude of striatal DA depletion and axonal pathology necessary to achieve motor deficits. This critical threshold may recapitulate what occurs in the clinic, where approximately ~70% of striatal DA and ~50% of nigral neurons are lost before motor symptoms are recognized [31]. Initial studies in rats utilizing chicken beta actin (CβA) or cytomegalovirus (CMV) vector promoters of serotypes 2 or 5 detected motor deficits in the cylinder task for forelimb akinesia and amphetamine-induced rotational behavior as early as 3 weeks post-α-syn injection. These behaviors, if observed, are weakly progressive [20,23,32]. Recent studies by Decressac and colleagues using rAAV6-α-syn with a synapsin promoter and woodchuck hepatitis virus posttranscriptional regulatory element (WPRE) in rats reported improved targeting and transduction of SNpc dopamine neurons with human wildtype α-syn resulting in reliable motor deficits [26]. This study was the first to demonstrate consistent, progressive behavioral deficits as well as marked striatal pathology prior to DA neuron death. 

In order to utilize the AAV α-syn overexpression model to develop novel therapies for PD a detailed understanding of the resulting pathology and motor impairments is required. In the present study we characterized nigrostriatal degeneration of varying vector titers (2.2 x 10^12^, 1.0 x 10^13^, 5.9 x 10^13^, 1.0 x 10^14^) using a rAAV2/5 vector construct with a CβA/CMV enhancer hybrid promoter to express human wildtype α-syn using unique two-site intranigral injection parameters. Two intranigral injection sites were chosen to optimize transduction throughout the entire rostrocaudal axis of the SN and rAAV2/5 was selected due to this serotype exhibiting trophism for nigral DA neurons [33]. We demonstrate that α-syn overexpression induces progressive SNpc DA neuron and neurite loss over a period of 8 weeks and that the magnitude of SNpc neuron loss is dependent on both vector titer and duration of α-syn expression. rAAV2/5-α-syn of the highest vector titer (1.0 x 10^14^ vector genomes/ml (vg/ml)) resulted in near complete SNpc DA neuron depletion over a period of 8 weeks. Our findings indicate that under conditions of seemingly equal levels of nigrostriatal degeneration, significant motor deficits are only observed following longer intervals of α-syn overexpression. Further, 8 weeks of α-syn overexpression resulted in significant deficits in ultrasonic vocalizations. These results establish a titer- and duration-dependent impact of rAAV2/5-mediated expression of human wildtype α-syn and allow for appropriate design and testing of future therapeutic neuroprotective strategies for PD.

## Materials and Methods

### Experiment 1: Characterization of a-syn-mediated toxicity following injection of rAAV2/5-α-syn of varying titers

Rats were unilaterally injected in the substantia nigra pars compacta (SNpc) with either 2.2 x 10^12^, 1.0 x 10^13,^ 5.9 x 10^13^, or 1.0 x 10^14^ rAAV2/5-human-α-syn, or rAAV2/5-green fluorescent protein (1.2 x 10^13^). Cohorts of rAAV2/5-α-syn injected rats were euthanized at 4, 8, or 12 weeks post-vector injection. rAAV2/5-GFP rats used as controls were euthanized 12 weeks post-vector injection. The efficiency of gene transfer (α-syn or GFP) was determined via α-syn immunohistochemistry or GFP autofluorescence. α-syn-mediated toxicity was examined using tyrosine hydroxylase (TH) immunohistochemistry and stereological analysis. 

### Experiment 2: The effect of rAAV2/5-α-syn mediated α-syn overexpression on TH immunoreactive (THir) nigral neuron survival, THir striatal terminals, and motor impairment

Rats were injected unilaterally in the SNpc with either 1.0 x 10^13^ or 1.0 x 10^14^ titer rAAV2/5-α-syn. Motor impairment was assessed one week prior to vector injection (baseline), and 4 (10^13^ and 10^14^ titers) or 8 (10^13^ titer only) weeks post-injection. All rats were tested in the bilateral tactile stimulation test, the cylinder test, and adjusting steps test. Additionally, ultrasonic vocalization recordings were collected in the 5.9 x 10^13^ titer rAAV2/5-α-syn injected group 8 weeks post vector injection as well as in naïve control rats of identical age. 1.0 x 10^14^ and 1.0 x 10^13^ titer injected rats were euthanized 4 or 8 weeks post-vector injection, respectively. All brains were processed for α-syn and TH immunohistochemistry. Stereological analysis of surviving THir neurons in the SNpc and striatal TH optical density measurements were completed to determine the extent of α-syn-mediated degeneration throughout the nigrostriatal system. 

### Animals

Male, Sprague Dawley rats (n = 62) and female, Sprague Dawley rats (n = 5, Harlan, Indianapolis, IN; 225-250g) were used in the current study. All animals were provided food and water ad libitum and housed in a reverse light-dark cycle conditions in the Van Andel Research Institute vivarium that is fully AAALAC approved. This study was specifically approved by the Institute for Animal Use and Care committee (IACUC) of Michigan State University. 

### Production of recombinant adeno-associated viral vectors

Production of the rAAV2/5-α-syn expressing vector was completed as previously described [34]. Briefly, human wild-type α-syn was cloned from human cDNA and inserted into an AAV plasmid backbone. The green fluorescent protein (GFP) control virus contained humanized GFP. The expression of the transgene for both vectors was driven by the chicken beta action/cytomegalovirus enhancer (CβA/CMV) promoter hybrid. Vectors contained AAV2 ITRs and were packaged into AAV5 capsids via co-transfection with a plasmid containing rAAV *rep* and *cap* genes and adenovirus helper functions. Particles were purified using iodixanol gradients and q-sepharose chromatography, and dotblot was used to determine vector titer [35]. In order to preserve viral stability and titer, viral preparations were never frozen and stored at 4°C. Viral preparations remained on wet ice during surgical procedures. All surfaces (pipettes, syringes, and microcentrifudge tubes) were coated in Sigmacote (Sigma-Aldrich, St. Louis, MO SL2) prior to coming in contact with the virus to minimize binding of viral particles. Four different rAAV2/5-α-syn titers were used in the current study: 2.2 x 10^12^, 1.0 x 10^13^, 5.9 x 10^13^, and 1.0 x 10^14^ vg/ml were used in Experiment 1, and 1.0 x 10^13^ vg/ml, and 1.0 x 10^14^ vg/ml were used in Experiment 2 with the exception that rats assessed for ultrasonic vocalizations received 5.9 x 10^13^ rAAV2/5-α-syn. The titer for the GFP control virus was 1.2 x 10^13^ vg/ml. 

### rAAV2/5-α-syn or GFP Injections

All surgical procedures were performed under isofluorane anesthesia (5% in O_2_ for induction and 2% in O_2_ for maintenance). Rats were placed in a stereotaxic frame and two 2 μl injections of either rAAV2/5-α-syn or rAAV2/5-GFP was injected in the left SN at coordinates (from dura) AP -5.3 mm, ML +2.0 mm, DV -7.2 mm, and AP -6.0 mm, ML +2.0 mm, and DV -7.2 mm. A Hamilton syringe fitted with a glass capillary needle (Hamilton Gas Tight syringe 80,000, 26s/2” needle; Hamilton, Reno, NV; coated in SigmaCote) was used for injection. The needle was lowered to the site and vector injection began immediately at a rate of 0.5 μl/minute and remained in place after the injection for an additional 5 minutes before retraction. 

### Behavioral Testing

In Experiment *2*, the extent of motor impairment was assessed 1 week prior to rAAV2/5-α-syn injection, and 4 weeks (1.0 x 10^14^ and 1.0 x 10^13^ injected rats) and 8 weeks (1.0 x 10^13^ injected rats) following rAAV2/5-α-syn injection. Tests performed included the cylinder test, the bilateral tactile stimulation test, and the adjusting steps test. Additionally, ultrasonic vocalization recordings were collected at 8 weeks post-vector injection in 5.9 x 10^13^ titer injected rats. 

#### Cylinder Test

This test of forelimb use asymmetry was performed as previously described [34,36]. Briefly, rats were placed in a clear plexiglass cylinder until 20 weight-bearing forepaw placements on the sides of the cylinder occurred, or until a maximum trial time of 5 minutes had elapsed. To determine if forepaw preference was present, the number of contralateral, ipsilateral, and simultaneous paw placements was recorded. Data are reported as the percentage of contralateral (to rAAV2/5-a-syn injection) forelimb use: [(contralateral + ½ both)/(ipsilateral + contralateral +both)] x 100. Rats with a unilateral nigrostriatal lesion will show a bias towards using the ipsilateral versus contralateral limb. 

#### Bilateral tactile Stimulation

This test was performed as previously described [37-39]. To test somatosensory asymmetry, each rat was individually removed from the home cage and an adhesive sticker (Tough-Spots ½” diameter, USA Scientific 9185-0504) was placed on the distal-radial aspect of both forepaws. Any cagemates were placed in a holding cage. Immediately after stimulus placement, the rat was returned to the home cage alone. The order and time to contact each stimulus was recorded with a maximum cutoff time of 60 seconds (s). If the rat did not contact the stimulus within 60s the sticker was then manually removed and a score of 60 was given for that forepaw. Five trials were performed on each rat. Faster contact times consistently on the ipsilateral forepaw indicate a somatosensory deficit in the contralateral forepaw. Data are presented as the average time to contact the *contralateral* forepaw within groups. For correlation figures data are presented as time to contact the *affected* forepaw for individual animals. 

#### Adjusting Steps Task

The adjusting steps or bracing test was performed as described previously [40,41]. To measure postural instability, stepping movements made with contralateral and ipsilateral forepaw were assessed. In this test, each rat was held by the torso with its hindlimbs and a single forelimb lifted above a table (36” wide) so that the weight of the rat’s body was supported by a single forepaw contacting the table. The experimenter moved the rat laterally over a fixed distance (36 inches) for a 10s period for 3 trials. Adjusting steps by each forelimb were recorded and averaged across trials. Rats with a unilateral nigrostriatal lesion make fewer adjusting steps with the forelimb contralateral to the injection site. Data are presented as the average number of adjusting steps made by the contralateral forepaw within groups and step number is reported for individual animals in correlation figures. 

#### Ultrasonic vocalization recording

Ultrasonic vocalizations (USVs) were examined at 8 weeks post-injection in rats that received 5.9 x 10^13^ rAAV2/5-α-syn (n = 11) and uninjected controls (n = 10). USVs from male rats were evoked using a sexual motivation paradigm [42-44]. In this paradigm each male rat was paired with a receptive female rat (in estrous) daily for several days until each male showed reliable sexual interest in the female which included sniffing, chasing, and attempted mounting behaviors. During this time, we observed that both males and females produce USVs in the 50 kHz range. For USV recording, a sensitive condenser microphone with a flat frequency response to 150 kHz and a working frequency response range of 10-180 kHz (CM16 + CMPA, Avisoft, Germany). Sampling rate was 214,174 Hz, 16-bits. The microphone was secured 15cm above the test cage and was attached to an ultrasound recording interface (UltraSound Gate 116 Hb; Avisoft Bioacoustics, Germany). A receptive female rat was placed in the home cage with one male rat until the male began to show signs of sexual interest. The female was then removed from the cage and USVs from only the male were recorded until at least 50 calls were produced. This typically required 1-5 minutes of recording depending on the rat. From these recordings acoustic parameters including duration (ms), bandwidth (Hz), intensity (dB), peak frequency (Hz), complexity, call rate, and latency to call were measured [45]. . 

#### Ultrasonic vocalization data analysis

Analysis of acoustic parameters was performed using Avisoft-SASlab Pro software (Avisoft Bioacoustics, Germany) according to previously described procedures [42,44]. Briefly, spectrograms were generated under a 512 FFT length and 75% overlap frame setup. Calls (50-kHz) were separated based on their complexity and divided into two general types of categories: simple and complex. The simple category included both simple and simple compound calls while complex included frequency modulated calls [42]. Call categorization was performed by a rater using visual and auditory inspection. The rater was masked to experimental condition. During inspection of calls all sounds determined to be noise and not USVs were removed if they interfered with the USV measurements. All other acoustic parameters were measured automatically using SASlab Pro. For each animal, the maximum, average, and average of top 10 highest values were calculated for duration, bandwidth, intensity, and peak frequency for both simple and complex calls. Call rate (all types of calls) was determined over the first 60 seconds of recording and call latency was scored as the time when the first call was made after the female was removed from the cage and recording commenced. 

### Tissue processing and immunohistochemistry

In Experiment *1*, rAAV2/5-α-syn or rAAV2/5-GFP injected rats were sacrificed at 4, 8 or 12 weeks following surgery. In Experiment *2*, 1.0 x 10^13^ and 1.0 x 10^14^ titer injected rats were euthanized at 8 or 4 weeks post vector injection, respectively. All animals were deeply anesthetized (60 m/kg, pentobarbital, i.p.) and perfused intracardially with 0.9% saline containing 1 ml/10,000 USP heparin. Brains were removed and post-fixed in 4% paraformaldehyde in 0.1 M/PO_4_ buffer for 7 days, then transferred to 30% sucrose in 0.1 M/PO_4_ buffer. Brains were frozen on dry ice and sectioned at 40 μm thickness using a sliding microtome. Immunohistochemical staining was performed using the free-floating method. 

#### α-syn immunohistochemistry for verification of transduction

Free-floating sections were blocked in 10% normal goat serum and incubated in primary antisera against α-syn (Invitrogen AHB0261, mouse anti-human α-syn, 1:2000) overnight at room temperature. Following primary incubation, sections were incubated in secondary antisera (Millipore AP124B, Goat anti-mouse, 1:400) for 2 hours. Antibody labeling was visualized using the Vector ABC detection kit with horseradish peroxidase (Vector Laboratories, Burlingame, CA) and exposure to 0.5mg/ml 3,3’ diaminobenzidine and 0.03% H_2_O_2_ in Tris buffer. Sections were mounted on subbed slides and coverslipped with Cytoseal (Richard-Allan Scientific, Waltham, MA). GFP sections were mounted on subbed slides, coverslipped with Vectashield HardSet Mounting Medium (Vector Laboratories H-1400), and transduction was visualized by native fluorescence using a Nikon 90i fluorescence microscope. 

#### Tyrosine hydroxylase (TH) immunohistochemistry for stereology

Sections containing the SN were blocked in 10% normal goat serum and incubated in primary antisera against TH (Millipore MAB318, mouse anti-TH, 1:4000) overnight at room temperature. Following primary incubation, TH-labeled sections were incubated in secondary antisera against mouse IgG (Millipore AP124B, Goat anti-mouse, 1:400) for 2 hours at room temperature, followed by the Vector ABC detection kit using horseradish peroxidase (Vector Laboratories, Burlingame, CA). Antibody labeling was visualized by exposure to 0.5 mg/ml 3,3’ diaminobenzidine and 0.03% H_2_O_2_ in Tris buffer. Sections were mounted on subbed slides and coverslipped with Cytoseal.

#### TH immunofluorescence for near infrared imaging and optical density analysis

Free-floating tissue sections were blocked in Odyssey blocking buffer (LI-COR Bioscience, Lincoln, NE, 927-40000) for 60 min at room temperature prior to primary antibody incubation for TH (Chemicon MAB318, Mouse anti-tyrosine hydroxylase, 1:1000, in Odyssey blocking buffer with 0.2% Triton-X) overnight at room temperature. Following primary incubation, tissue was incubated in secondary antisera for 2 hours at room temperature (LI-COR Biosciences 926-32210, IRDye 800CW Goat anti-mouse, 1:250 in Odyssey blocking buffer). Sections were then rinsed in 0.1 M Tris-buffered saline and immediately mounted onto subbed slides, dehydrated, and coverslipped with Cytoseal. Slides were then imaged using the Odyssey infrared image system (LI-COR Bioscience) (800 nm channel, 42 μm resolution) to examine TH expression in the nigrostriatal system (see Densitometry). 

#### α-syn and TH immunofluorescence for co-expression within nigral DA neurons and detection of aggregates

Free-floating sections were blocked in 10% normal goat serum and incubated in primary antisera against TH (Millipore AB152, rabbit anti-TH, 1:4000) overnight at room temperature. Following primary incubation, sections were incubated secondary antibody (Invitrogen A-11037, Goat anti-rabbit Alexa Fluor 488, 1:400) for 2 hours at room temperature. Sections were rinsed in Tris-buffered solution and re-blocked in 10% normal goat serum. Sections were then incubated in in primary antisera against α-syn (Invitrogen AHB0261, mouse anti-human α-syn, 1:2000) overnight at room temperature followed by incubation in secondary antibody (Invitrogen A-11032, Goat anti-mouse Alexa Fluor 594, 1:400) for 2 hours at room temperature. Sections were mounted on subbed slides and coverslipped with Vectashield HardSet Mounting Medium. Immunolabeling was visualized using an Olympus FluoView® FV10i confocal laser scanning microscope. 

### Stereology

Assessment of the total number of TH immunoreactive neurons in the substantia nigra was completed as previously described [34]. Briefly, stereology was performed using a Nikon Eclipse 80i microscope (Nikon), StereoInvestigator software (Microbrightfield Bioscience, Williston, VT) and Retiga 4000R camera (QImaging, Surrey, BC Canada). Using the optical fractionator principle, THir neurons α-syn injected and control nigral hemispheres in every sixth section of the entire SN were counted at 60X magnification. A coefficient of error < 0.10 was accepted. Data are reported as the average percent of intact THir neurons remaining within groups.

### Densitometry

Serial sections were fluorescently labeled for TH and slides were viewed on a LI-COR Odyssey near-infrared scanner (LI-COR Biosciences). To identify unilateral loss of TH protein, integrated signal intensities were collected in both the lesioned and intact THir striatal hemispheres through the entire striatum on normalized slides. Additionally, because SNpc neuron projections target the dorsolateral striatum, THir measurements were collected in the dorsolateral region. To define the dorsolateral region, the striatum was hemisected in half vertically, and a horizontal line was drawn across from the base of the lateral ventricle, leaving the pie-shaped wedge along the corpus callosum as the dorsolateral region. Slides were normalized by analyzing and subtracting background staining intensity from the contralateral cortex for each animal. The average of the raw integrated intensity values (arbitrary units) was calculated for each animal to normalize for disparities in number of sampling sites between animals. 

### Statistical Analysis

All statistical tests were completed using SigmaPlot software (version 11.0, Systat Software, Inc., San Jose, CA). Survival of THir neurons in Experiment *1* was confirmed by a one-way ANOVA analysis with a single treatment factor. Holm-Sidak *post hoc* analysis was used to determine significance within treatments. Values are presented as the mean percent of intact nigral neurons ± SEM. Differences in THir neuron survival and TH expression in the striatum between 1.0 x 10^13^ and 1.0 x 10^14^ rAAV2/5-α-syn injected rats were determined by a two-way repeated measures ANOVA using titer and lesion status factors. Holm-Sidak *post hoc* analysis was used to determine significance within titers. USVs were compared between naïve control and 5.9 x 10^13^ titer rAAV2/5-α-syn rats using a one tailed Student’s t-test. One way repeated-measure ANOVAs followed by Holm-Sidak *post hoc* analyses were conducted to confirm the presence of functional deficits. In Experiment *2*, correlation analysis was performed using the non-linear regression and straight line fit equations. Statistical significance was set at *p* < 0.05. 

## Results

### α-syn expression in the nigrostriatal system after rAAV2/5 injection

**Figure 1:**
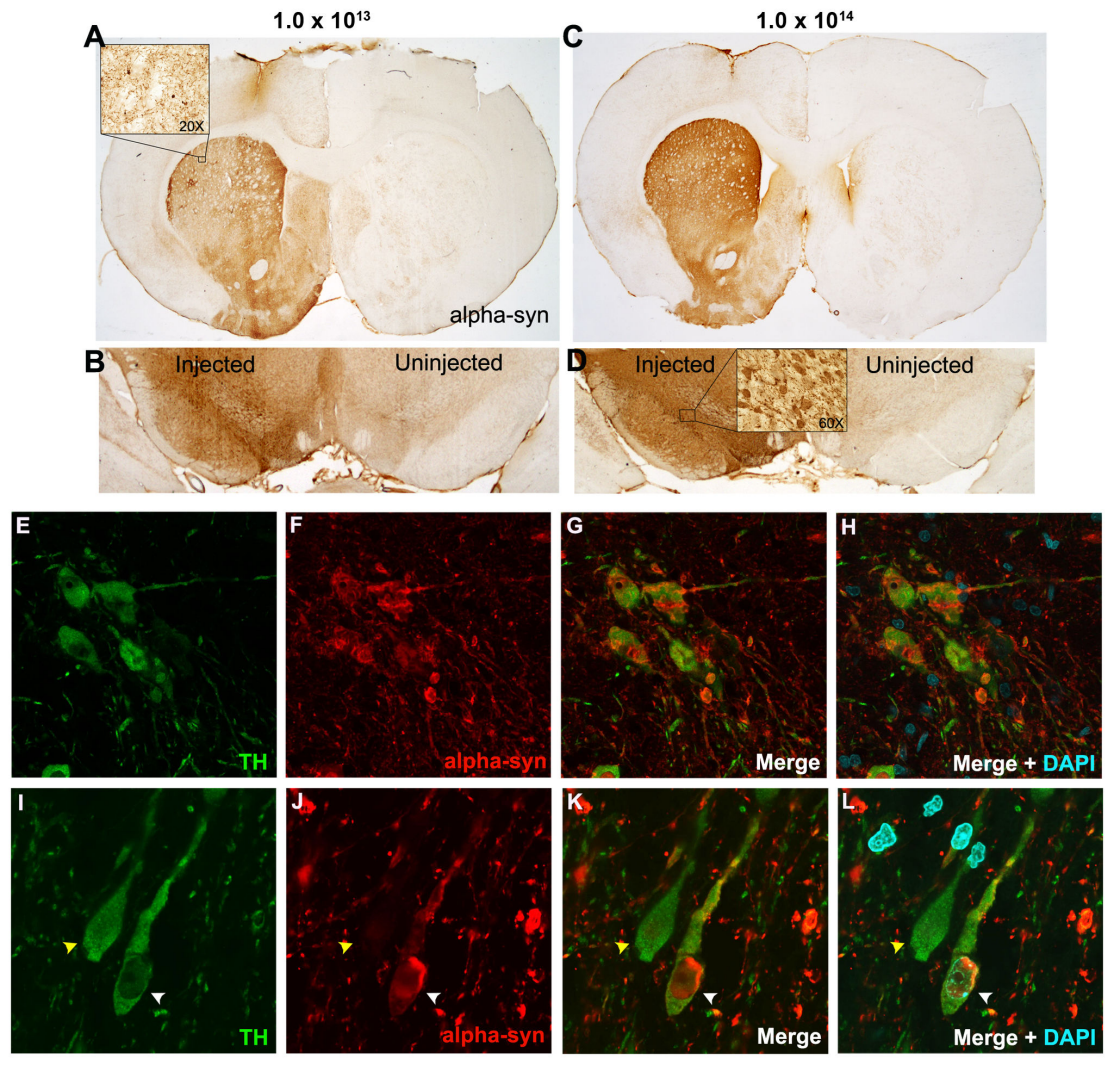
Human α-syn expression in the striatum and SN following intranigral rAAV2/5-α-syn injection. Representative samples of human α-syn immunoreactivity in rat striatum (**A**, **C**) and substantia nigra (**B**, **D**) following intranigral injection with rAAV2/5-α-syn. **A**-**B**. Transduction following injection of 1.0 x 10^13^ vg/ml titer rAAV2/5-α-syn. (**A**) α-syn immunoreactivity is observed throughout the striatum ipsilateral to injected nigra. Insert shows striatal α-syn positive neurites and protein aggregates (20X). (**B**) α-syn immunoreactivity observed in the injected SN. **C**-**D**. α-syn expression following injection of 1.0 x 10^14^ vg/ml titer rAAV2/5-α-syn. α-syn immunoreactivity in the ST (**C**) and SN (**D**) following vector injection. Insert shows α-syn positive nigral neurons at 60X. **E**-**L**. α-syn co-expression and aggregation within SNpc THir neurons. **E**-**H**. Lower magnification image of α-syn immunoreactive aggregation within THir neurons and surrounding neurites. **I**-**L**. THir neurons with (white arrow heads) and without (yellow arrow heads) α-syn accumulation at 168x.

We examined the expression of α-syn in the striatum and substantia nigra at 4, 8 or 12 weeks following 2.2 x 10^12^, 1.0 x 10^13^, 5.9 x 10^13^, or 1.0 x 10^14^ intranigral rAAV2/5-α-syn injection. At all time points, immunodetection of α-syn revealed abundant immunoreactivity of wild type human α-syn in the injected SNpc and SNpr and surrounding areas of the midbrain (Figure 1B, D). Immunofluorescent co-localization of α-syn and TH revealed that α-syn was present within a significant proportion of SNpc DA neurons. α-syn appeared to fill the cytoplasm and neurites and intraneuronal aggregates within TH immunoreactive (THir) neurons in the SN were frequently observed (Figure 1E-J). Immunodetection of α-syn throughout the ipsilateral striatum after intranigral injection indicated that α-syn protein was transported anterogradely to fill striatal dopaminergic terminals (Figure 1A, C). α-syn-ir dystrophic neurites appeared swollen with the frequent presence of large α-syn-ir aggregates that were confined to the striatal hemisphere ipsilateral to vector injection (Figure 1A). 

### α-syn-mediated SNpc THir neuron degeneration is dependent on vector titer

**Figure 2:**
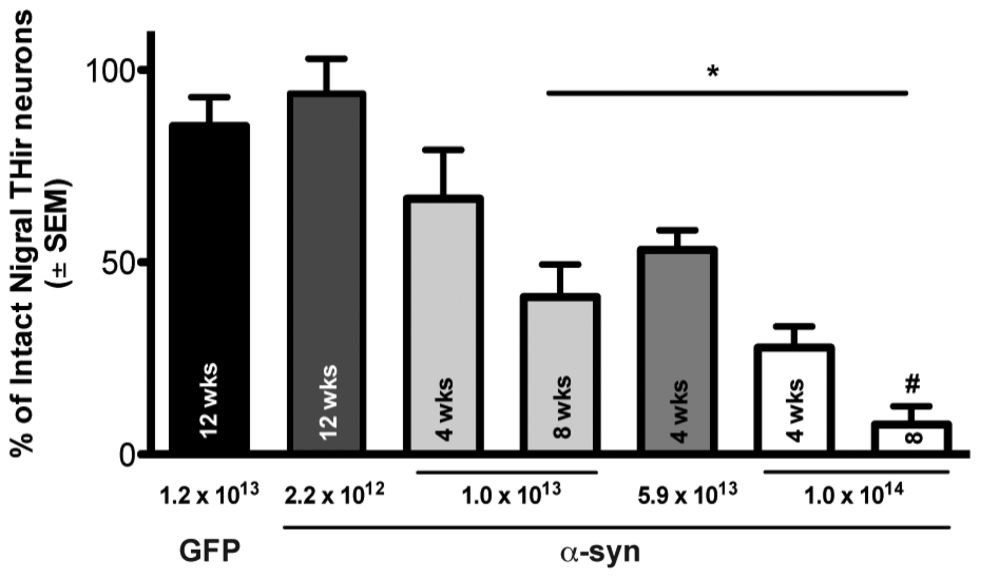
α-syn-mediated neurotoxicity in the SNpc is titer-dependent. Stereological quantification of surviving THir neurons in rats injected with 1.2 x 10^13^ vg/ml rAAV2/5-GFP or 2.2 x 10^12^, 1.0 x 10^13^, 5.9 x 10^13^, or 1.0 x 10^14^ vg/ml rAAV2/5-α-syn. Rats were euthanized at 4, 8, or 12 weeks as indicated. Injection of 1.2 x 10^13^ rAAV2/5-GFP or 2.2 x 10^12^ rAAV2/5-α-syn did not result in significant THir neuron loss over the 12 week post-injection interval. THir neurons were significantly decreased eight weeks after injection with 1.0 x 10^13^ rAAV2/5-α-syn, but not after 4 weeks, when compared to both 1.2 x 10^13^ rAAV2/5-GFP and 2.2 x 10^12^ rAAV2/5-α-syn (*, p < 0.03). Injection of 1.0 x 10^14^ titer rAAV2/5-α-syn resulted in significantly fewer THir neurons after eight weeks than either 1.0 x 10^13^ titer rAAV2/5-α-syn or 5.9 x 10^13^ rAAV2/5-α-syn after 4 weeks (*, p ≤ 0.02). No significant difference were observed between 1.0 x 10^13^ rAAV2/5-α-syn after 8 weeks of expression, 5.9 x 10^13^ rAAV2/5-α-syn after 4 weeks or 1.0 x 10^14^ rAAV2/5-α-syn after 4 weeks (p ≥ 0.05).

To determine if α-syn overexpression resulted in the degeneration of THir neurons in the SNpc, rats in Experiment *1* were injected unilaterally in the substantia nigra with 2.2 x 10^12^, 1.0 x 10^13^, 5.9 x 10^13^, or 1.0 x 10^14^ rAAV2/5-α-syn, or 1.2 x 10^13^ rAAV2/5-GFP (transduction control). α-syn vector-injected rats were sacrificed 4, 8, or 12 weeks post-rAAV2/5-α-syn injection (Figure 2). Control animals were sacrificed 12 weeks post-rAAV2/5-GFP injection. GFP expression over 12 weeks produced a small, non-significant reduction in SNpc THir neurons, with rats maintaining an average of ~85% THir neurons in the ipsilateral injected SNpc compared to the contralateral hemisphere (*p* > 0.05). Significant THir neuron degeneration also did not occur in 2.2 x 10^12^ rAAV2/5-a-syn titer injected rats twelve weeks following injection with ~94% of THir neurons maintained (p > 0.05). In addition, four weeks following injection of the 1.0 x 10^13^ titer rAAV2/5-α-syn a non-significant ~35% loss was observed compared to GFP controls (*p* > 0.05). In contrast, eight weeks after injection of 1.0 x 10^13^ rAAV2/5-α-syn, significant loss of THir SNpc neurons (~60%) was observed compared to rats injected with 2.2 x 10^12^ rAAV2/5-α-syn over 12 weeks or rats injected with rAAV2/5-GFP (*F*_(6,17)_ = 12.347, *p* < 0.03). Similarly, four weeks after injection with rAAV2/5-α-syn of 5.9 x 10^13^ titer significant THir neuron loss (~50%) was observed when compared to either the rAAV2/5-GFP group (p = 0.023) or the 2.2 x 10^12^ rAAV2/5-α-syn group (p = 0.007). Rats sacrificed at both 4 and 8 weeks following injection with 1.0 x 10^14^ rAAV2/5-α-syn displayed the most pronounced depletion of THir SNpc neurons (~70% 4 weeks, ~90% eight weeks) compared to both the rAAV2/5-GFP and the 2.2 x 10^12^ rAAV2/5-α-syn group (p < 0.001). Further, 8 weeks following surgery rats injected with the highest 1.0 x 10^14^ titer of rAAV2/5-α-syn possessed significantly fewer THir neurons than the number of THir neurons observed 4 weeks following injection of 5.9 x 10^13^ titer rAAV2/5-α-syn (p < 0.02). *Post hoc* analysis in a one-way ANOVA revealed that although decreases in THir neuron survival were observed within the 1.0 x 10^13^ and 1.0 x 10^14^ titers between 4 and 8 weeks, this decrease did not reach significance (*p* > 0.05). Lastly, and important for the design of Experiment *2*, there was no significant difference between the extent of SNpc THir neuron loss observed at 4 weeks following 1.02 x 10^14^ rAAV2/5-α-syn injection and 8 weeks following 1.02 x 10^13^ rAAV2/5-α-syn injection (*p* > 0.05). These results are depicted in Figure 2. 

**Figure 3:**
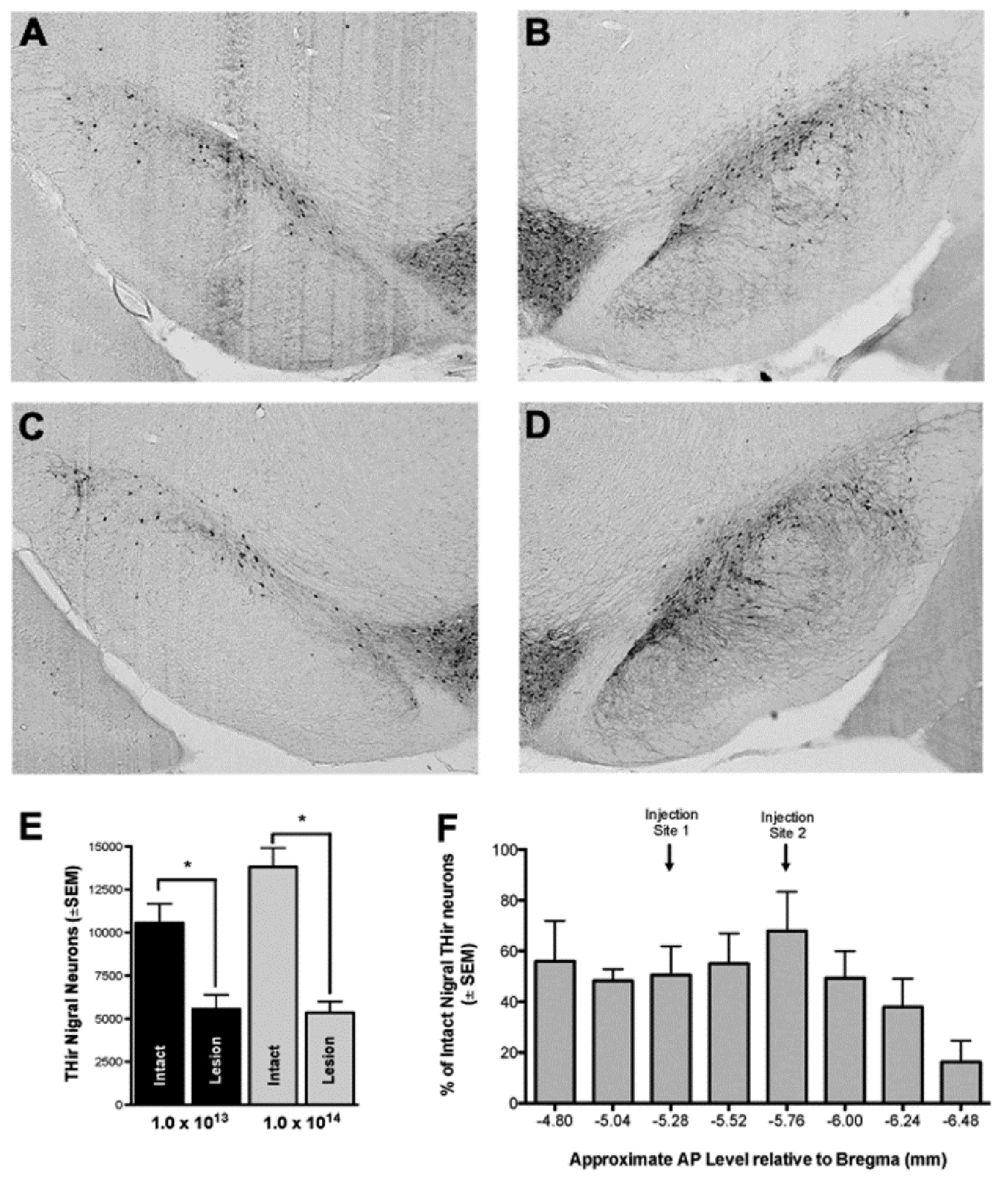
rAAV2/5-α-syn mediated neurotoxicity in the SNpc (Experiment 2). **A**, **C**. Surviving THir neurons in the α-syn overexpressing lesioned SNpc of 1.0 x 10^13^ vg/ml (A) and 1.0 x 10^14^ vg/ml (**C**) titer vector injected rats. **B**, **D**. THir neurons in the naïve, intact SNpc of 1.0 x 10^13^ (**B**) and 1.0 x 10^14^ (**D**) titer vector injected rats. **E**-**F**. α-syn overexpression resulted in significant THir neuron degeneration in both the 1.0 x 10^14^ and 1.0 x 10^13^ titer vector injected groups (*p ≤ 0.005) and magnitude of degeneration was not different between titer groups (**F**). **G**. Magnitude of THir neuron loss was equal across the SNpc. Percent survival of THir neurons in the SNpc of each of the eight sections demonstrates that degeneration occurred throughout the SNpc. Vector injection sites were -5.28 and -5.76 mm relative to bregma.

Experiment *2* was conducted to further characterize the impact of α-syn overexpression on degeneration of SNpc DA neurons, depletion of THir terminals in the striatum and motor deficits. In addition, Experiment *2* sought to determine whether duration of α-syn overexpression/DA depletion was a factor in striatal terminal degeneration or motor performance. To accomplish this, two different vector parameters resulting in a statistically identical magnitude of nigrostriatal degeneration (60-70%) were selected based on stereological assessment of THir SNpc neurons in Experiment *1*. Thus, rats were injected with 1.0 x 10^13^ or 1.0 x 10^14^ titer rAAV2/5-α-syn and euthanized at 8 weeks or 4 weeks, respectively. A two-way repeated measures ANOVA revealed a main effect for treatment (*F*_(1,23)_ = 63.759, *p* < 0.001), revealing that in both the 1.0 x 10^14^ and 1.0 x 10^13^ titer rAAV2/5-α-syn groups, the α-syn injected SNpc possessed a significantly fewer number of surviving THir neurons then the contralateral uninjected SNpc (Figure 3A-F). Higher 1.0 x 10^14^ titer rAAV2/5-α-syn injected rats had an average of ≅ 39% THir neurons remaining at 4 weeks post injection in the lesioned SNpc compared to contralateral SNpc (*p* < 0.001). At 8 weeks post 1.0 x 10^13^ titer rAAV2/5-α-syn injection, rats had an average of ≅ 53% remaining THir neurons in the lesioned SNpc (*p* = 0.002). As predicted by the results of Experiment *1*, there were no significant differences between the 1.0 x 10^14^ titer 4 week and 1.0 x 10^13^ titer 8 week treatment groups (*p* > 0.05, Figure 3E).

To determine whether SNpc degeneration occurred throughout the rostrocaudal axis of the SNpc or was localized to sites of rAAV2/5-α-syn injection (- 5.28 and - 5.76 mm relative to bregma), stereological analysis was used to count the number of surviving THir neurons within eight individual SN sections along the rostrocaudal axis from rats in both the 4 week 1.0 x 10^14^ titer and 8 week 1.0 x 10^13^ titer rAAV2/5-α-syn treatment groups, corresponding to a full series of tissue when cut in a 1 in 6 series. Estimated populations for each section from the ipsilateral and contralateral SNpc were averaged across all animals and percent survival was calculated. THir neuron loss ranged from ~33-84% along the rostrocaudal axis of the SNpc, with rostral and mid regions on average maintaining ≅ 50% of THir neurons and increased degeneration occurring the in the caudal aspect of the SNpc. Beginning in the rostral region of the SNpc, ~55% of THir neurons were maintained at – 4.80 mm from bregma, ~ 48% at -5.04 mm, ~ 50% at – 5.28 mm, ~ 55% at – 5.52 mm, ~ 68% at – 5.76 mm, ~ 50% at – 6.00 mm, ~ 38% at -6.24 mm, and ~ 16% at – 6.48 mm. These results are depicted in Figure 3F. 

### α-syn overexpression results in reduced TH immunoreactivity in the striatum

**Figure 4:**
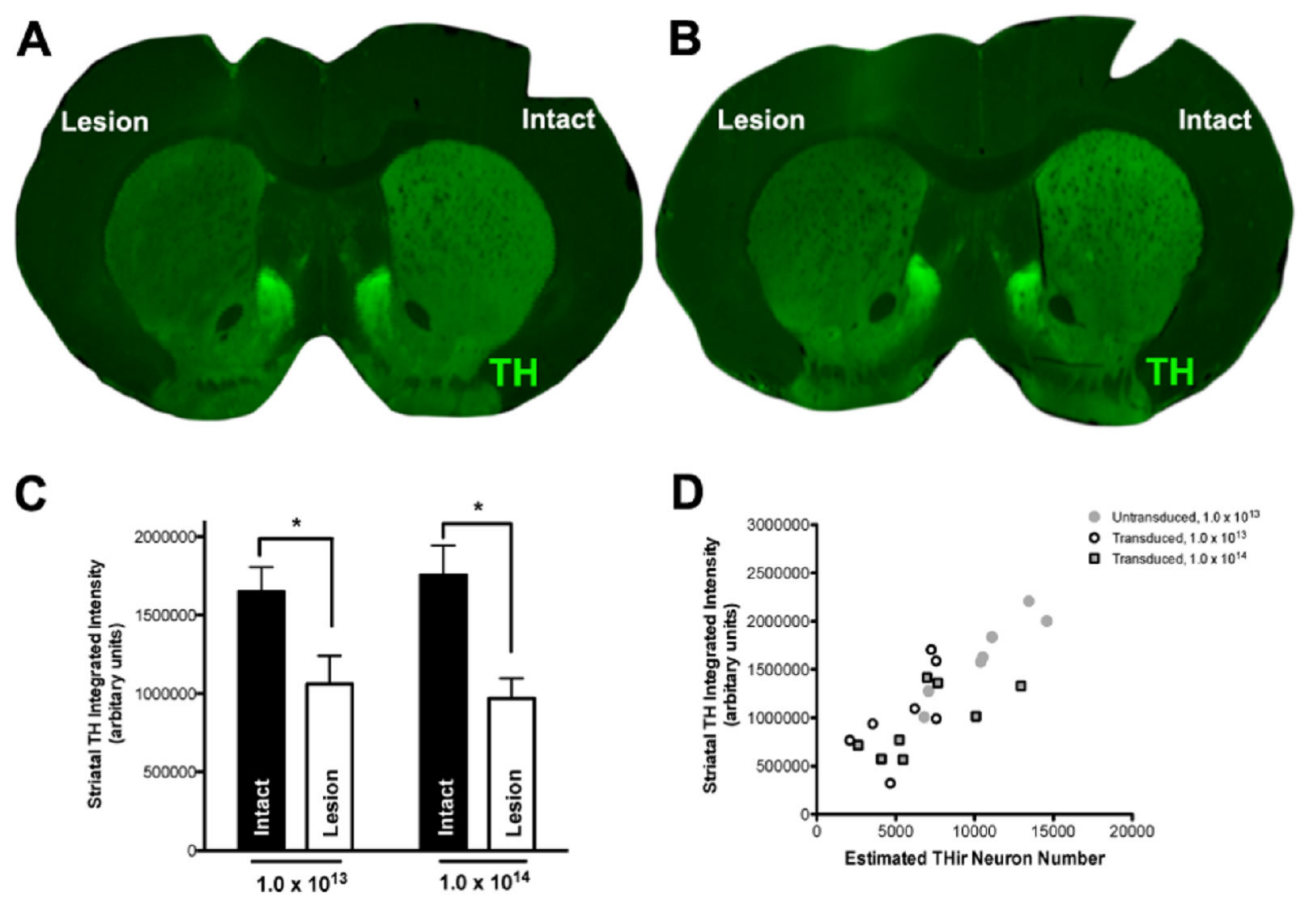
α-syn overexpression decreased striatal TH expression. **A**-**B**. Pseudocolored near infrared TH immunofluorescence in the α-syn overexpressing (lesion) and intact striatum of 1.0 x 10^13^ (vg/ml) titer (**A**) and 1.0 x 10^14^ (vg/ml) titer (**B**) injected rats. **C**. TH expression was significantly reduced in the α-syn overexpressing striatum (*p < 0.001).

Near infrared integrated intensity measurements of THir in serial sections were collected from the striatum in animals from Experiment *2*. α-syn overexpression produced a significant loss of THir in the striatum ipsilateral to SN injection (*F*_(1, 23)_ = 22.407, *p* < 0.001), with no significances observed different between the 4 week 1.0 X 10^14^ and 8 week 1.0 x 10^13^ titer rAAV2/5-α-syn injected groups. On average, striatal THir was reduced by ≈ 42% in the α-syn-overexpressing striatum compared to the contralateral striatal hemisphere (Figure 4A-C). 

### α-syn overexpression produces functional deficits in forelimb use

**Figure 5:**
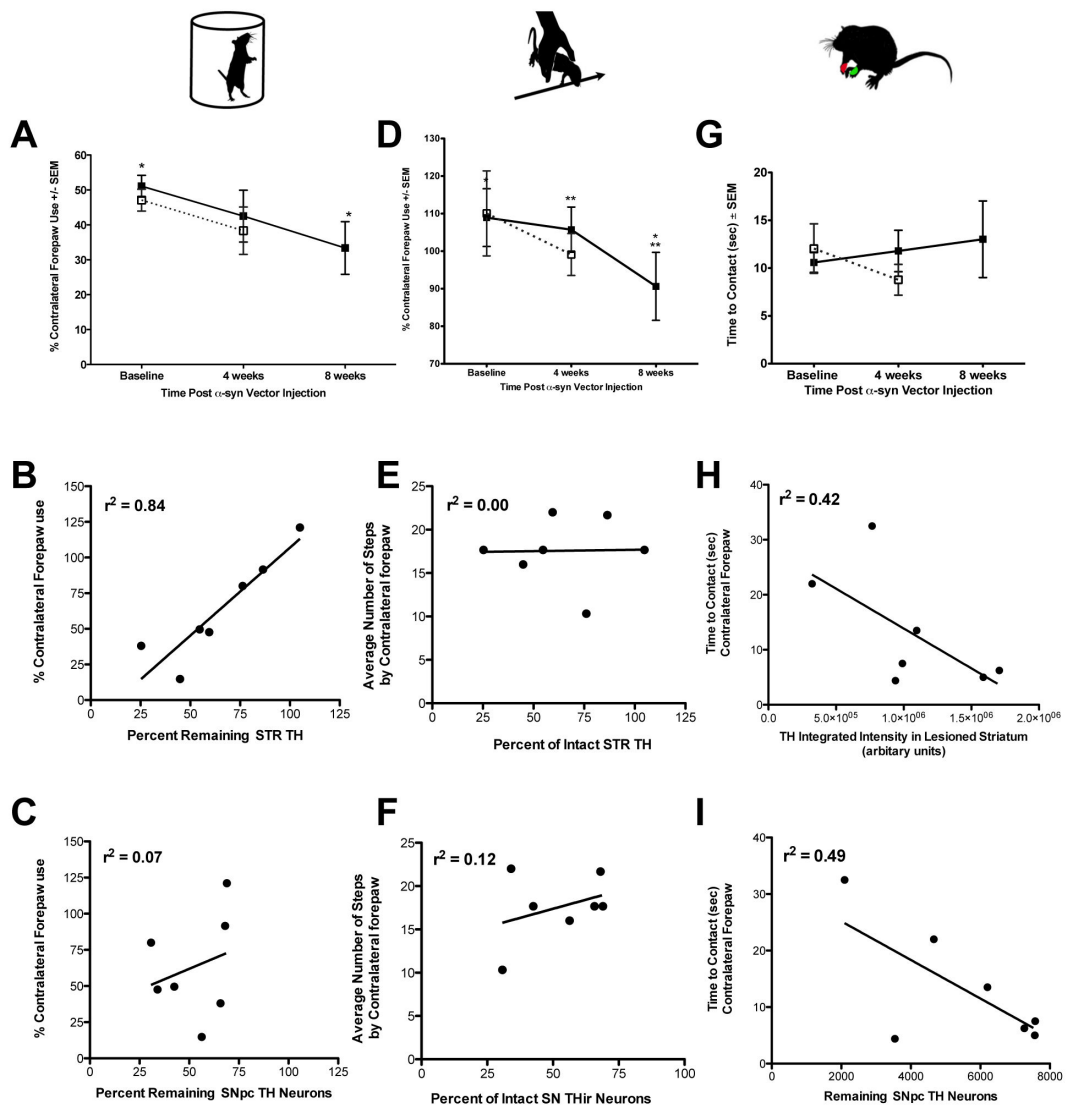
α-syn overexpression-mediated forelimb motor impairments are duration-dependent. **A**-**C**. Cylinder test; (**A**) 1.0 x 10^13^ vg/ml titer rAAV2/5-α-syn injected rats (■) displayed significant deficits in contralateral forepaw use at 8 weeks post vector injection compared to baseline (*, *p* = 0.045). 1.0 x 10^14^ vg/ml injected rats (☐), with equivalent SNpc THir neuron degeneration, displayed no significant deficits at 4 weeks post-vector injection (*p* > 0.05). **B**-**C**. Forelimb use was significantly correlated with TH expression in the lesioned striatum (**B**, r^2^ = 0.84, p = 0.004), but not with surviving THir neuron numbers in the lesioned SNpc (**C**). **D**-**F**. Adjusting steps task; 1.0 x 10^13^ titer rAAV2/5-α-syn vector injected rats experienced significant reductions in contralateral forepaw use over 8 weeks (*p = 0.008, **p = 0.036) while 1.0 x 10^14^ titer injected rats did not (*p* > 0.05). **E**-**F**. Number of adjusting steps taken by the affected forepaw did not correlate with striatal THir expression or numbers of surviving THir nigral neurons. **G**-**I**. Bilateral tactile stimulation; no differences in time to contact the affected forepaw were observed in either titer group (*p* > 0.05) (**G**). **H**-**I**. The time to contact the affected forepaw was only moderately correlated to striatal THir expression (**H**, r^2^ = 0.42) and numbers of surviving SNpc THir neurons (**I**, r^2^ = 0.49).

Next, we examined the impact of α-syn overexpression on forelimb sensorimotor function in 4 week 1.0 x 10^14^ and 8 week 1.0 x 10^13^ titer rAAV2/5-α-syn injected rats. 1.0 x 10^14^ titer rats were tested prior to rAAV2/5-α-syn injection and 4 weeks post-vector injection. 1.0 x 10^13^ titer injected rats were tested before rAAV2/5-α-syn injection, 4, and 8 weeks post-vector injection. Motor performance was assessed using the cylinder test for forelimb akinesia, the adjusting steps task, and the bilateral tactile stimulation test. A decrease in contralateral forelimb use was observed in the 8 week 1.0 x 10^13^ titer rAAV2/5-α-syn treatment group in both the cylinder and adjusting steps tests (Figure 5A, D). A one-way repeated measures ANOVA revealed a significant deficit in contralateral forepaw use (*F*_(2,19)_ = 4.072, *p* = 0.045) in the cylinder test for 8 weeks 1.0 x 10^13^ titer rAAV2/5-α-syn injected rats between baseline and 8 week time points (*p* = 0.043). Similarly, a one-way repeated measures ANOVA reveled a significant deficit in contralateral forelimb use (*F*_(2,18)_ = 5.310, *p* = 0.024) in the adjusting steps task test for 1.0 x 10^13^ titer rAAV2/5-α-syn injected rats between baseline and 8 week time points (*p* = 0.030). No significant motor deficit was present in the 4 week 1.0 x 10^14^ titer rAAV2/5-α-syn injected treatment group between baseline and 4 weeks in these two tests (*p* > 0.05). Ipsilateral and contralateral contact times did not significantly differ between groups. There was no impairment in the bilateral tactile stimulation task in either the 8 week 1.0 x 10^13^ or 4 week 1.0 x10^14^ titer rAAV2/5-α-syn injected treatment groups (Figure 5G). These results are depicted in Figure 5A-I. 

### Relationship between nigrostriatal depletion and forelimb impairments

α-syn overexpression resulted in degeneration of both THir SNpc neurons and striatal neurites in our model. We examined if the behavioral deficits observed at 8 weeks following 1.0 x 10^13^ titer rAAV2/5-α-syn injection were correlated with either SNpc THir neuronal loss or TH immunoreactivity in the striatum. The relationship between percent of contralateral forepaw use and remaining THir neurons in the lesioned SN or TH expression in the striatum in 1.0 x 10^13^ titer rAAV2/5-α-syn injected rats at 8 weeks was examined by non-linear regression. Regression analysis revealed that the extent of striatal TH loss was significantly correlated with forelimb use in the cylinder test (*r* ^2^ = 0.84, *F*_(1,5)_ = 25.4376, *p* = 0.004, Figure 5B). Forelimb use was not significantly correlated with striatal TH loss in the bracing test, or with the percentage of intact SNpc THir neurons in the cylinder or bracing tests (*p* = n.s., Figure 5B, C, E, F). For the bilateral tactile stimulation test, the relationship between time to contact the impaired forepaw and SNpc THir neuron counts or measurements of striatal TH intensity were examined. Moderate, but insignificant correlations were observed between the numbers of remaining SNpc THir neurons in the lesioned SN (*r* ^2^ = 0.49, *p* = n.s.) or TH expression in the lesioned striatum (*r* ^2^ = 0.42, *p* = n.s.) with time to contact the contralateral forepaw (Figure 5H, I). 

### α-syn overexpression produces deficits in ultrasonic vocalizations

**Figure 6:**
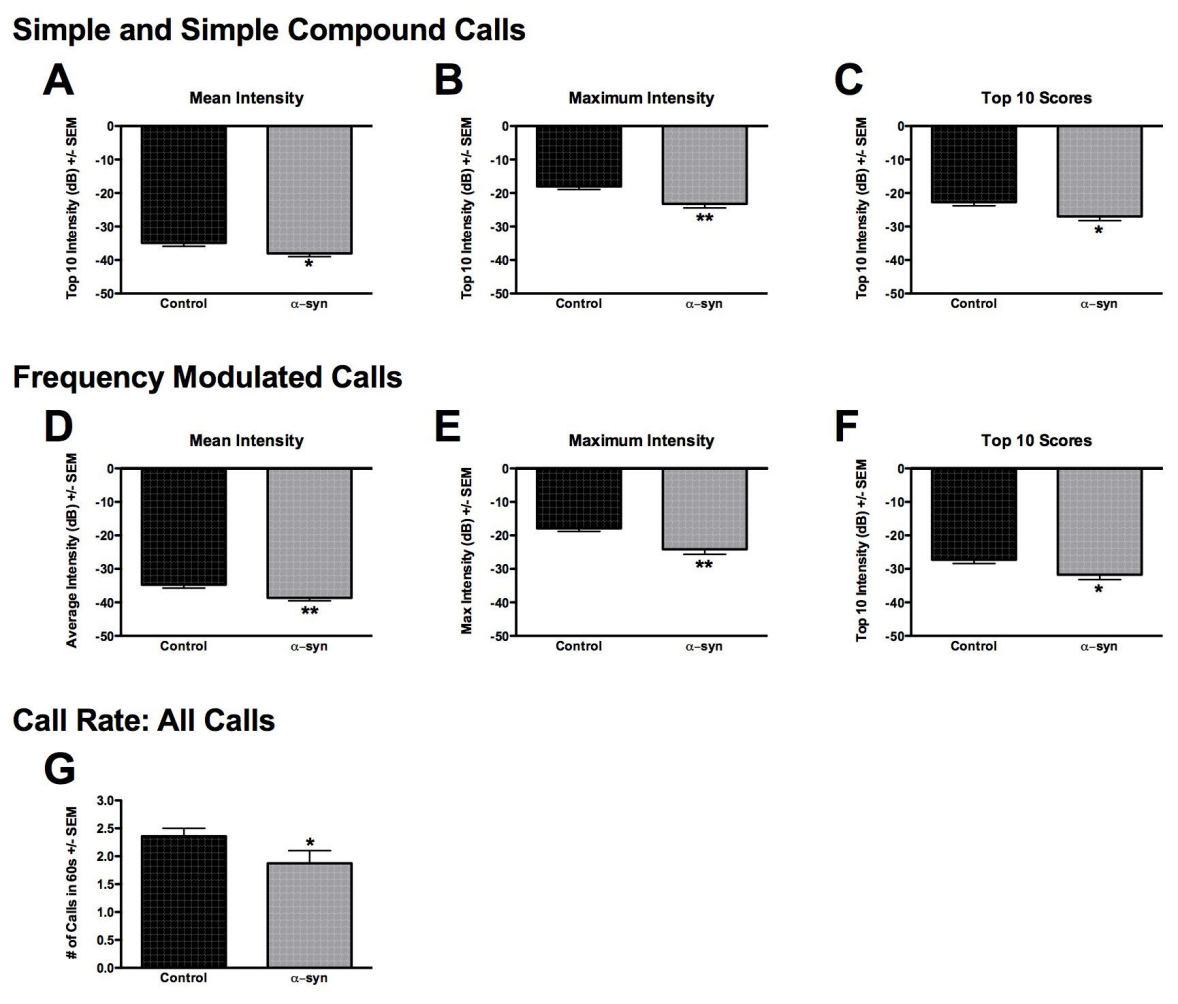
α-syn overexpression leads to deficits in ultrasonic vocalizations. Analysis of the mean, maximum and the top 10 simple and simple compound type calls (**A**-**C**) as well as frequency modulated calls (**D**-**F**) revealed that α-syn overexpression significantly reduced call intensity (*, *p* < 0.05). Further, call rate for all calls over the first 60 seconds of recording was also significantly reduced in rAAV2/5-α-syn rats compared to control rats (**G** *, p* < 0.05).

USVs elicited from naïve control rats and rats injected with 5.9 x 10^13^ titer rAAV2/5-α-syn rats (8 weeks) were analyzed for the following acoustic parameters: duration, bandwidth, intensity, and peak frequency for both simple and complex calls, call rate, and latency to call. There were no significant differences in call type, duration, bandwidth, or peak frequency between naïve control and rAAV2/5-α-syn rats (Tables 1 and 2). However, call intensity was significantly reduced in both simple (max- *t*_(19)_ = 3.35, *p* < 0.01; mean- *t*_(19)_ = 2.27, *p* < 0.05; Top 10- *t*_(19)_ = 2.56, *p* < 0.01, Figure 6A-C) and frequency modulated calls (max- *t*_(19)_ = 3.51, *p* < 0.01; mean- *t*_(19)_ = 2.98, *p* < 0.01; Top 10- *t*_(19)_ = 2.39, *p* < 0.05, Figure 6D-F) in rAAV α-syn rats compared to naïve controls. Call rate over the first 60 seconds of recording was also significantly reduced in rAAV2/5-α-syn rats compared to control rats (*t*_(19)_ = 1.79, *p* < 0.05, Figure 6G). Latency to call did not differ between control and rAAV2/5-α-syn rats (*p* > 0.05).

**Table 1:** Acoustic parameters of frequency modulated calls in naïve control and rAAV α-syn rats.

**Parameter**	**Naïve Control**	**rAAV α-syn**
Complex calls (%)	62.95±2.71	69.30±3.22
*Duration (ms)*		
Max	0.12±0.016	0.08±0.012
Mean	0.04±0.003	0.04±0.002
Top 10	0.06±0.003	0.05±0.005
*Bandwidth (Hz)*		
Max	36530±2390	34373±1612
Mean	15714±947	17871±981
Top 10	24785±2072	25844±1817
*Peak Frequency* (*Hz*)		
Max	74870±970	78636±1742
Mean	62093±835	65549±1295
Top 10	66780±1058	71244±1953

**Table 2:** Acoustic parameters of simple calls in naïve control and rAAV α-syn rats.

**Parameter**	**Naïve Control**	**rAAV α-syn**
Simple calls (%)	37.05±2.71	30.70±3.22
*Duration (ms)*		
Max	0.09±0.005	0.12±0.011
Mean	0.04±0.001	0.04±0.002
Top 10	0.06±0.003	0.07±0.003
*Bandwidth (Hz)*		
Max	36730±2674	33964±2600
Mean	12312±750	11660±1105
Top 10	29689±2066	26437±2489
*Peak Frequency* (*Hz*)		
Max	74170±1126	76618±2091
Mean	58851±585	59691±1341
Top 10	68628±1091	70047±1767

## Discussion

Our results demonstrate that utilizing two-site nigral injections of rAAV2/5 to overexpress wildtype human α-syn results in the progressive death of dopaminergic nigral neurons and significantly reduces THir in the striatum. We confirm that nigral degeneration is directly related to vector titer and that longer durations of α-syn expression appear to also increase the magnitude of degeneration. α-syn overexpression resulted in significant impairments in contralateral forelimb use in both the cylinder and adjusting steps task and deficits in USV call intensity and call rate. Characteristic α-syn immunoreactive inclusions were observed in SNpc THir neurons and were most prevalent in dorsolateral aspects of the striatum.

In the present study we made statistical comparisons between the level of nigral toxicity resulting from rAAV α-syn of titers ranging from 2.2 x 10^12^ – 1 x 10^14^ and rAAV GFP with a titer of 1.2 x 10^13^. Expression of GFP over a 12 week period did not produce significant THir neuron degeneration. However, in some cases our GFP vector titer was lower than our α-syn vector titer. Therefore, one limitation of our study is that we cannot rule out a contribution of non-specific toxicity with rAAV α-syn titers higher than 1.2 x 10^13^. Future studies will need to directly examine this issue. Nonetheless, due to the fact that significant nigral degeneration was observed eight weeks following 1.0 x 10^13^ rAAV α-syn we can directly link expression of α-syn protein to the toxicity observed in this particular cohort of rats. 

In AAV-α-syn overexpression models, the presence and extent of motor impairments has been highly variable across studies. This is probably due to the preservation of substantial levels of striatal DA, with the majority of studies only achieving moderate degeneration of nigral DA neurons and striatal terminals [20,23,26,46]. Previous studies have reported behavioral impairments in the cylinder task, adjusting steps task, corridor tests, and amphetamine-induced rotations, but only under conditions in which severe degeneration of 60-80% was achieved [26,29]. AAV-α-syn-induced nigrostriatal degeneration in our study was approximately ≈ 50% with motor impairments observed in the cylinder and adjusting steps tasks. The cylinder task has been shown to be a sensitive indicator of DA loss in this model, as deficits have been observed as early as 5 weeks post-vector injection, when ~40% of striatal TH and ~50% of nigral neurons have degenerated [26,29]. As anticipated, the level of striatal TH immunoreactivity highly correlated to cylinder task performance, as has been previously reported in this model of PD utilizing slightly different vector and surgical parameters [29]. Performance deficits were also observed in the adjusting steps test. Prior studies indicate that in the rAAV-α-syn model, a critical threshold of 50% striatal DA and nigral neuron loss must occur before seeing deficits in this test [20,29,46]. Contralateral forepaw use in the adjusting steps task was only moderately correlated to striatal TH levels in our study. It is possible, should more significant degeneration be achieved (more than 50% loss), that a stronger corollary relationship may exist between α-syn-mediated denervation and contralateral paw use in the adjusting steps task. However, in a recent study where α-syn overexpression-mediated degeneration reached almost 70% of THir nigral neurons and striatal neurites, the number of adjusting steps by the contralateral forepaw was also only moderately correlated with striatal TH levels [29]. Together, these results suggest that although the adjusting steps task can reveal the presence of motor deficits, the cylinder test is the more reliable measure to predict the level of DA depletion in the rAAV-α-syn model.

α-syn overexpression-mediated sensorimotor deficits appeared to be dependent on the interval between rAAV2/5-α-syn injection and behavioral assessment. Specifically, despite seemingly equivalent levels of nigral and striatal degeneration resulting from 4 weeks of α-syn overexpression via the 1.0 x 10^14^ titer rAAV2/5 injections and 8 weeks of α-syn overexpression resulting from 1.0 x 10^13^ titer rAAV2/5-α-syn injections, behavioral impairments were only observed in the 8 week 1.0 x 10^13^ titer rAAV2/5-α-syn treatment group. These behaviors likely result from the culmination of α-syn mediated DA terminal dysfunction or alterations in post-synaptic striatal elements triggered by DA depletion, or both. Prior studies have established progressive loss of striatal dopaminergic neurites and decline in function of DA release machinery and related motor impairments following α-syn overexpression [9,26,29]. DA depletion results in the post-synaptic remodeling in the target striatum including reductions in the density of dendritic spines on medium spiny neurons and time-dependent increases in postsynaptic D2 receptors [47,48]. Although not examined in the present study, it is likely that both these pre- and postsynaptic phenomena underlie the disparity in the behavioral results. Increased terminal dysfunction and postsynaptic alterations would be expected to occur over an 8 week period compared to the 4 week period, causing motor deficits that can not be explained by morphological assessment alone.

In addition to sensorimotor impairments, cranial sensorimotor deficits were also detected in this model using USV recording and analysis methods [44,49] Rodents produce USVs, which are analogous to human vocalizations in several ways including serving a communicative function [50-53] and being produced via aggressive airflow through the larynx [54,55] In PD, dysarthria, which includes vocal deficits such as reduced loudness and pitch variability, and a vocal tremor [56], are common and can severely impact the quality of life for patients by impairing communication [57]. USV deficits in the classic 6-hydroxydopmine model of PD are reminiscent of the voice deficits observed in patients [44,49]. A recent study showed that USV deficits manifest following nigrostriatal dopamine cell loss in mice with broad overexpression of human wildtype α-syn (unpublished data). The present study is the first to demonstrate that targeted unilateral nigrostriatal α-syn overexpression also results in significant deficits in aspects of USVs, specifically call intensity and call rate. To date, similar deficits in call intensity have been observed in aged rats, unilateral 6-hydroxydopamine rats, and α-syn overexpressing mice [44]. USV deficits in the rAAV2/5-α-syn model will be a useful outcome measure in therapeutic studies. 

Results from the current study, together with prior findings, illustrate the importance of characterization of the neuropathology and behavioral impact in distinct rAAV-α-syn overexpression models. Although nigrostriatal degeneration, appearance of a-syn immunoreactive aggregates, and behavioral deficits are consistent between rAAV-α-syn models, variations in vector construction and injection parameters will influence the extent of nigrostriatal degeneration in each model. Our data identify that two months of human wildtype α-syn overexpression resulting from two intranigral injections of rAAV2/5-α-syn (1.0 x 10^13^ vg/ml) produce a significant degeneration of approximately 50% SNpc DA neurons along the rostral-caudal axis of the SNpc. Further, this rAAV-α-syn overexpression paradigm yields an average 40% reduction in striatal TH levels that is highly correlated with contralateral forelimb performance in the cylinder task. This rAAV2/5-α-syn model characterization will provide a critical framework in which to test PD therapeutics in the future. 
